# The Value and Present Status of Vaginal Cesarean Section1Read before the meeting of the Tri-state Medical Society at Toledo, Ohio January, 8, 1908.

**Published:** 1908-06

**Authors:** M. Stamm

**Affiliations:** Fremont, Ohio


### The Value and Present Status of Vaginal Cesarean Section.[1]

By M. STAMM, M. D., Fremont, Ohio.

[*Detroit Medical Journal, April, 1908*]

DUHRSSEN, of Berlin, brought this operation before the medical profession about thirteen years ago (*Allgemeine Deutsche Aerztezeitung,* April 1st, 1895), and theoretically laid down its rules and indications which seem to have suffered very little change by time and experience. At first a number of theoretical objections were raised against this operation and the profession was slow in giving it a practical test. With increased experience, however, these objections have been gradually removed. An additional reason for its tardy acceptance may be found in the introduction, at that time, of new dilators by Bossi and others. A sufficient number of operations have now been made in Europe, especially in Germany, and within the last four years in this country, to enable us to speak of its merits and its various indications. The proof that it has some abiding qualities and that it is accepted as a safe and practical procedure in obstetric surgery, is demonstrated by the fact that a certain Berlin school which, up to a few years ago, had no words of recommendation for this operation, now shows a disposition to vindicate its priority to one of its own adherents.

In studying the literature upon this subject very little doubt will be in the mind of the reader that Duhrssen is not only the

---

1. Read before the meeting of the Tri-state Medical Society at Toledo, Ohio January, 8, 1908.

intellectual, but also the practical originator of this operation. It is true that Acconci, of Italy, has been credited with having performed the first vaginal cesarean section on July 4, 1895; a closer study, however, ought to convince the unbiased reader that to Duhrssen belongs the undisputed priority in this question. Acconci's case was operated for carcinoma of a pregnant uterus. After the interior and posterior incisions were made into the cervix he still had to resort to dilators to get sufficient access to the fetus, a step which Duhrssen's method, if properly carried out, will never require. Moreover, his case died and such incidents are generally not of a nature to entice others to repeat such an operation very soon. His case was not published before October, 1896, and it is evident from his report that, at that time, he had no idea of the importance and wide applicability of vaginal cesarean section, as not a word was said about its possible value and its different indications, besides, his first claim of priority was not brought forth before 1898. Duhrssen demonstrated that the incised uterus, after having been emptied of its contents, can be sutured and united again and perform its former function, also that we can empty a gravid uterus at any time and save the child, provided it is viable. Acconci naturally extirpated the cancerous uterus. It would seem that in questions of priority the maxim laid down by Fritsch, when he spoke of Trendelenburg's position, could be accepted as a fair and just one. On that occasion he said: "The priority must be vindicated to the one who has succeeded in making an invention or discovery common property, who establishes its reason and its indications and recommends it to such an extent that it will be universally accepted." Animated by such a spirit, I think, no one will successfully contest Duhrssen's priority.

The purpose of vaginal cesarean section is to empty a pregnant uterus at once through the vaginal route by an anterior or posterior incision, or both combined, into the cervix and lower segment of the uterus, without opening the peritoneal cavity, in cases where the cervix is totally or partially closed or where there is absence of pain or any effort on the part of the uterus to expell the fetus in due time. Duhrssen in his earlier publications laid down the following indications:

(1) Abnormal conditions of the cervix or lower segment of the uterus (carcinoma, myoma, rigidity or stenosis of the cervix and partial pouch-like distention of the lower uterine portion.)

(2) Dangerous conditions of the mother which may be removed or relieved by prompt emptying of the uterus (affections of the heart, lungs and kidneys.)

(3) Conditions of the mother where death is imminent and can be foreseen.

The last two conditions have value only in cases where the cervix is closed and not dilatable or where the depressing influence of labor pain should be obviated, as in affections of the heart and lungs. In pregnancy complicated with cancer of the uterus he advocated immediate vaginal cesarean section with subsequent extirpation of the uterus, no matter at what time of pregnancy or at what stage of labor his condition is encountered. Since that he has extended these indications also to the child where its life is threatened. Objection has been raised from different sides in regard to the title of "vaginal cesarean section," to which Duhrssen has not failed to answer (*Volkmann's Klin. Vortrage,* p. 232.) He then refers to Plimus where cesarean section received its name from the fact that the child was delivered "a caeso matris utero," or from the incised uterus of a pregnant woman. Duhrssen says: "If then we make an incision per vaginam into the uterus of a pregnant or parturient woman so that the child can be delivered through a previously closed cervix, the name 'vaginal cesarean section' is, in my opinion, not a specially bad one, and it has been adopted by Fritsch, Schauta and others without comment." As Duhrssen recommends in most cases an anterior and posterior incision, I think myself, the name "anterior and posterior vaginal hysterectomy" would not be any essential improvement over the above title.

Technic: Duhrssen's first description (1896), seems not to differ much from the technic he follows at present, and has described in his monograph (*"Der Vaginale Kaiserschnitt,"* Berlin, 1884.) I may be permitted to give you a translation in substance: after having emptied the bowels and bladder and disinfected the vulva, its vicinity and the vagina thoroughly, a right, lateral vagino-perineal incision will relieve the resistance of the lower third of the vagina in case of a primipara. If the levator ami muscle is divided by this incision a large closed fist can be readily introduced into the vagina and the vaginal vault and portio vaginalis can be brought to view by short, broad specula. The vaginal portion is then seized on either side with bullett forceps, which may be immediately supplanted by two silk tractors (Fadenzugel) and the posterior lip is split sagitally up to the vaginal junction. In extending this incision the posterior vaginal vault is severed about 4 cm. and the Douglas peritoneum is pushed away from the posterior wall of the cervix and uterine body by means of a speculum introduced into this opening. In a similar manner the anterior os and vaginal vault is raised and from this incision the vaginal wall is separated frcm the bladder by a few strokes of the scissors.

To facilitate this manipulation the vaginal wall may be severed from the cervix by a transverse incision of about 2 cm. and then the bladder and peritoneal fold are loosened from the anterior wall of the cervix and body of the uterus. By this procedure the anterior and posterior uterine walls are exposed fully to the extent of 6 cm. and are rapidly split with scissors, first the posterior and then the anterior wall. The opening thus made, and into which the amniotic sac will fall at once if the membranes are still intact, must be large enough to readily admit the fist of a strong man. This hand will promptly reach for one foot and extract the child. If the uterus contracts well, which may be aided by hypodermic injections of ergotine before the operation, you may wait for spontaneous separation of the placenta and then deliver it by expression. In case of uterine atony, however, the placenta may be detached by the hand and tamponade of the uterus may eventually be resorted to after Duhrssen's method. This latter procedure may prove very simple since the large aperture in the lower uterine segment will admit good sized specula so that large quantities of gauze can be quickly introduced between them into the uterine cavity. For this purpose and for the subsequent suture of the several parts the silk tractors, which transfixed the cervix from the start, serve nicely, as by sufficient traction the vaginal and uterine incisions can be brought down to the vaginal introitus. In this way the posterior incision of the uterine wall can conveniently be closed without the aid of a speculum by through and through catgut sutures tied upon the side of the mucous membrane of the cervix. We proceed about in the same manner with the anterior incision, except that the sutures are tied upon the anterior instead of the inner surface of the uterus. The vaginal incisions are united by continuous catgut sutures, leaving only a small opening close to the uterus. It is important to introduce small gauze strips through them into the ante- and retrouterine cavity for drainage to prevent retention of blood and secretion. These strips and uterine tampon are removed after three to twenty-four hours. In case a vagino-perineal incision was made the operation will end with its closure. The vaginal wall is united by continued catgut suture and the perineal cut by deep silk worm sutures. The latter are removed in from eight to ten days. As a rule the child is delivered in about six minutes and the whole operation finished in from twenty-five to thirty minutes. The after treatment does not differ from the one followed in normal labor; the subjective condition after operation is as favorable as that found in any easy, spontaneous confinement.

Duhrssen speaks of a radical and conservative vaginal cesarean section. In the radical operation the womb is removed, i. e., for cancer or sepsis. This operation does not differ much from the method generally adopted for the extirpation of the cancerous uterus. The uterus is best split into halves before the ligaments are tied, as there is less danger from hemorrhage. In the conservative operation the uterus, after having been sutured, is left in its place and in a condition to carry on its function as before. So far it has been performed upon the following indication:

Uncontrollable vomiting, combined with cicatricial rigidity of the cervix; stenosis after amputation for prolapse; heart affection and stenosis; rigidity of the cervix, tetanus uteri; painful edema of the posterior portion; dangerous hemorrhage due to premature detachment of placenta; mitral stenosis, patient was moribund; heart disease and nephritis, acute dilatation, circumscribed edema (Clark); chorea gravidarum; sepsis; pouch-like distention of the anterior uterine wall; incarcerated retroverted uterus; torpidity of the uterus after induced abortion, narrow pelvis (2 cases); overdistended and attenuated lower uterine segment; placenta previa; dead child, long, hard contracted cervix; lack of pain in very young or old primipara; that pelvis where the fetal heart became weak and os was not dilated, head high up, version could be more readily made after incision.

In the majority of cases of vaginal cesarian section, Duhrssen's advice to make an anterior and posterior incision should be heeded, as there is less danger of laceration of the uterus or bladder and the incisions do not have to be made so deep as would be necessary with a single incision. In my first paper on this subject I advised to make the longest incision in the portion that presents itself best, having done so in my first case. This has been sanctioned by Duhrssen. Schauta, in one case, made the posterior incision exclusively. In case of narrow vagina, Duhrssen strongly advised the vagino-perineal incision, known as Schuchardt's method. In my second case the passing of the head made a tear into the vagina up to the parametrium; this was brought together by continued catgut suture and healed by primary union. The placenta, of course, should always be removed before the sutures are tied. Ruhl advises to dilate the cervix with metal dilators before the operation as in that way retention of secretion or lochia might be obviated. I think a small iodoform gauze strip will do it as well, if not better, and in my hands I did not find it to interfere with the sutures. In atony of the uterus gauze tamponade is especially indicated. Hammerschlag, however, mentions a case which had eight convulsions before the birth of the child, six hours after delivery patient was seized again with about eleven convulsions, with tracheal rales, temperature 104 degrees, pulse 132, and deep coma. He finally came to the conclusion that the uterine tampon might produce irritation and keep up the convulsions. The latter ceased at once after removal of the tampon and the temperature went down to 99 degrees and pulse to 96 per minute, recovery was undisturbed.

On theoretical grounds some fear of excessive hemorrhage has been entertained by men of limited or no experience, but so far no such accident has been recorded. To avoid this it is well to make the incision strictly in the median line, as the venous plexus on both sides of the cervix furnish the chief source of bleeding. There are two cases of sepsis reported out of about 140 cases; from this it appears that the danger from such a source is not any greater than that of any other obstetric operation. Moreover, in these cases manual or mechanical dilatation has preceded the cesarean section, so it would be difficult to decide whether such interference or the operation should be made responsible for it. If the uterus is already septic the danger is not as great in the vaginal as in the abdominal cesarean section, as in the former the abdominal cavity is not opened and there is a better chance for drainage by leaving the uterine and vaginal cuts unsutured. Having to deal with such a case of septic infection we may, under proper circumstances and after taking all the features of the case into account, remove the source of infection at once by extirpation of the uterus immediately after the delivery of the child and placenta. A successful case of Doederlein is reported by Baisch Hegar's Beitrage Bd 6 Heft 3.) Patient 39 years old, XI gravid., six months pregnant, with prolapse and elongation of the cervix, decubitus and incipient sepsis. Cervix would admit one finger, anterior and posterior Douglas was opened, incision in the lower half of both parametria, the anterior wall of the cervix was split, perforation and extraction of the fetus, total separation of the parametria, closure of the abdominal cavity.

Most of the cases which have been examined some time after operation revealed such slight cicatricial changes that they would have escaped the attention of the examiner had he been aware of the fact that such an operation had been performed. There are, however, some cases reported where parts were not fully united. As to the effect of vaginal cesarean section upon subsequent labor Duhrssen, Wennerstrom, Ruehl, Liepman, Jerie and myself have reported about 11 cases. A case of Duhrssen was delivered three years later, with balloon dilatation of six hours duration, of a living child. Wennerstrom's case had spontaneous labor two years after operation. My second case was operated on September 6, 1903. She gave birth to a child 7½ months old on July 15, 1904, before any physician could reach her and without any untoward effect. Both mother and child are in good health today. The question has repeatedly been asked, what advantage has the vaginal cesarean over the ventral section? Experience so far shows that the former is less dangerous which, no doubt is due in great part to the fact that the peritoneal cavity is not opened. Moreover, I think patients, or at least their friends, will sooner consent to a vaginal operation than to abdominal cesarean section, the mention of which is still apt to create in the mind of the public a certain amount of consternation.

It is advisable to make this operation in a well equipped hospital, if possible. A man skilled in such work, however, should not hesitate to operate in private houses in case of emergency. My first case was operated on at the residence of the patient, about 16 miles from home. Ruehl, of Germany, and Miller, of New Orleans, also report one case each operated on at patient's home.

